# Preparation, toxicity reduction and radiation therapy application of gold nanorods

**DOI:** 10.1186/s12951-021-01209-4

**Published:** 2021-12-28

**Authors:** Lina Xie, Xujia Zhang, Chengchao Chu, Yingqi Dong, Tianzi Zhang, Xinyue Li, Gang Liu, Wen Cai, Suxia Han

**Affiliations:** 1 Department of Radiation Oncology, The First Affiliated Hospital, Xi’an Jiaotong University, Xi’an, 710061 Shaanxi China; 2 Institute of Medical Engineering, School of Basic Medical Sciences, Health Science Center, Xi’an Jiaotong University, Xi’an, 710061 Shaanxi China; 3 Eye Institute of Xiamen University, Fujian Provincial Key Laboratory of Ophthalmology and Visual Science, School of Medicine, Xiamen University, Xiamen, 361102 Fujian China; 4 State Key Laboratory of Molecular Vaccinology and Molecular Diagnostics Center for Molecular Imaging and Translational Medicine, School of Public Health, Xiamen University, Xiamen, 361102 Fujian China

**Keywords:** Gold nanorods (GNRs), Preparation, Toxicity reduction, Radiation sensitization, Radiation therapy

## Abstract

Gold nanorods (GNRs) have a broad application prospect in biomedical fields because of their unique properties and controllable surface modification. The element aurum (Au) with high atomic number (high-Z) render GNRs ideal radiosensitive materials for radiation therapy and computed tomography (CT) imaging. Besides, GNRs have the capability of efficiently converting light energy to heat in the near-infrared (NIR) region for photothermal therapy. Although there are more and more researches on GNRs for radiation therapy, how to improve their biocompatibility and how to efficiently utilize them for radiation therapy should be further studied. This review will focuse on the research progress regarding the preparation and toxicity reduction of GNRs, as well as GNRs-mediated radiation therapy.

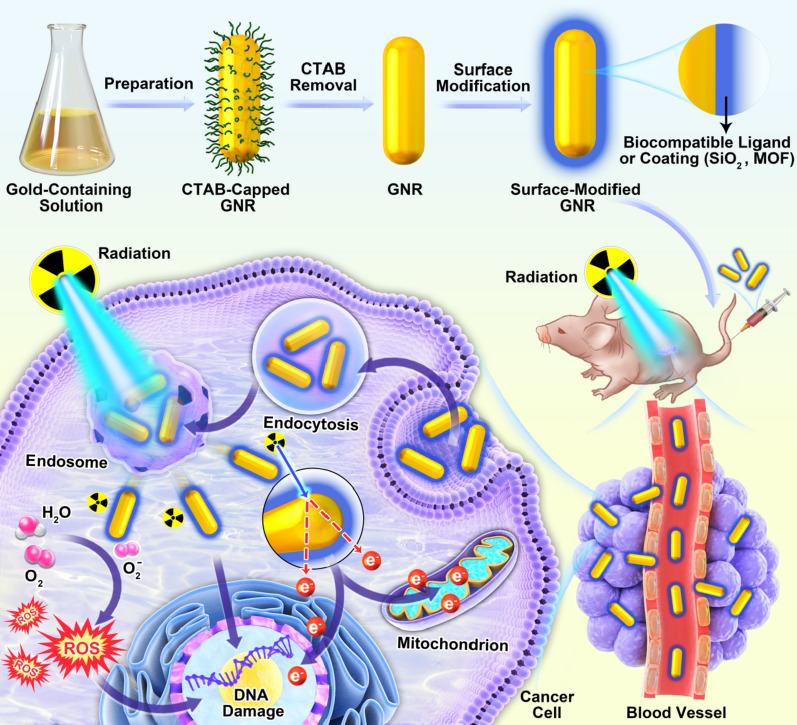

## Introduction

Malignant tumors are one of the main causes of death in humans; radiation therapy is a local treatment modality for tumors, and more than 50% of patients with cancer need radiation therapy [1]. Radiation includes not only external radiation (X-rays, electron lines, etc.) generated by various types of machines or accelerators outside the body but also internal radiation (eg. alpha- and beta rays) generated by radioisotopes inside the body. The ionization effect caused by radiation can kill the tumor cells by directly destroy cellular DNA, or indirectly react with water molecule to produce reactive oxygen species (ROS) to damage DNA or other cellular components [1]. The role and status of radiation therapy in tumor therapy have become increasingly prominent and this method has emerged as one of the main means to treat malignant tumors; however, the radiation resistance of tumor cells remains a problem. As a type of ionizing radiation-responsive agents, radiosensitizers are commonly used in clinical practice to improve the effect of radiation therapy; however, traditional radiosensitizers, such as platinum-based drugs, show high toxicity and side effects and low selectivity to tumor cells. Therefore, radiosensitizers that can effectively target tumor tissues with good biocompatibility should be developed.

The fast development of nanotechnology applied in biomedicine has opened a new way for the diagnosis and treatment of tumors. Nanomaterials show passive- and active tumor targeting ability via the enhanced permeability and retention (EPR) effect and their surfaces modified with targeting molecules, respectively [2, 3]. Nanomaterials with high atomic number (high-Z) have unique characteristics of photoelectric decay, and they have shown good potential in tumor radiation therapy as radiosensitizers. Many problems that restrict tumor radiation therapy may be overcome, and new opportunities may be explored to promote the further development of radiation therapy by introducing nanomaterials as radiosensitizers or sensitizer carriers into radiation therapy [4, 5].

Gold nanoparticles (GNPs), as high-Z materials (Z = 79), show various morphologies such as sphere-shaped, rod-shaped, nanocage, and nanostar, and they have been widely explored in biomedicine [6, 7]. Gold nanorods (GNRs) belong to a type of GNPs; they have unique optical properties, good biocompatibility, easy-to-control surface modification, and have been extensively investigated in bioimaging, drug and gene delivery, biosensors, and cancer treatment [8, 9]. GNRs show two absorbance maxima corresponding to the transverse surface plasmon resonance (TSPR) and longitudinal surface plasmon resonance (LSPR); the position of TSPR is near 520–530 nm and it does not change with the aspect (length/diameter) ratio of GNRs, but the position of LSPR can be tuned in the near-infrared (NIR) region (600–900 nm) by adjusting the aspect ratio of GNRs [10]. When GNRs are irradiated with a laser, photon energy interacts with the lattice, vibration intensifies, temperature increases, and ROS form in the presence of molecular oxygen, that is, photothermal- and photodynamic effects are produced; this property enables GNRs to be involved in the synthesis of a variety of GNRs-based nanocomposites for photothermal therapy [8, 11–17]. GNRs also elicit a photoacoustic (PA) effect, as such, they are considered a promising PA contrast agent [8, 18]. Moreover, GNRs can be utilized for computed tomography (CT) imaging [8, 19] and radiation sensitization because of their strong X-ray absorption ability.

**Fig. 1:**
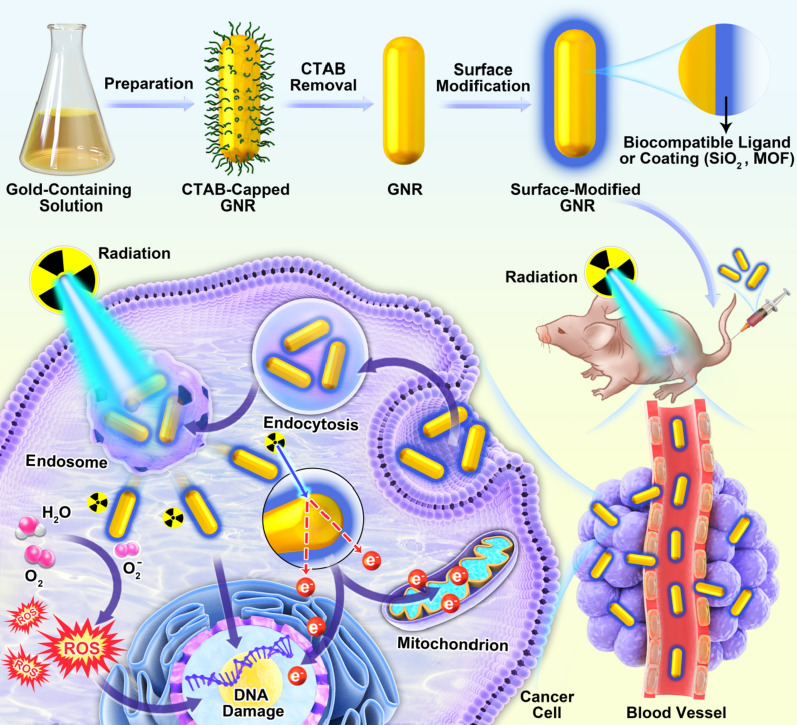
Preparation, toxicity reduction and radiation therapy application of GNRs

As high-Z materials, GNRs are potential radiosensitizers for radiation therapy [5–7]. In addition, GNR’s excellent NIR photothermal property can help achieve the goal of reducing the radiation dose to reach the same radiation therapy efficacy. Before GNRs are applied to radiation therapy, their toxicity must be considered. To the best of our knowledge, there are very few reports regarding the research progress on the toxicity reduction and radiation therapy application of GNRs. In this manuscript, the research progress on the preparation, toxicity reduction, as well as radiation therapy application of GNRs are discussed (Fig. 1).

## Preparation of GNRs

The preparation methods for synthesizing GNRs have progressed rapidly. The commonly used routes for preparation of GNRs include seed-mediated growth-, seedless-, template-, photochemical-, electrochemical- and sonochemical method; these routes have shown great advantages in the synthesis of GNRs with good dispersity and particle uniformity.

### Seed-mediated growth method

**Fig. 2:**
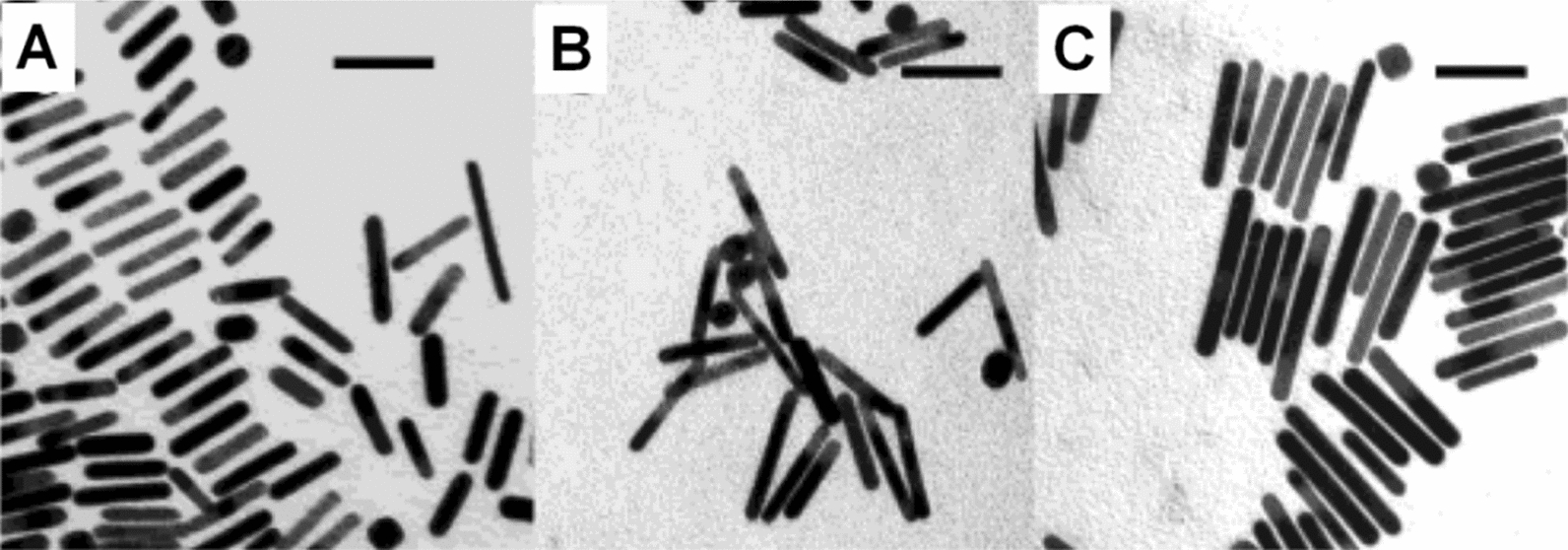
Transmission electron microscopy (TEM) images of GNRs (prepared by seed-mediated growth method using binary surfactant mixture) with plasmon band energy at **A** 880, **B** 1130, and **C** 1250 nm, respectively. The scale bar is 50 nm (Reproduced with permission [23]. Copyright 2003, American Chemical Society)

Seed-mediated growth method was first introduced by Brown et al. [20]; it is relatively simple and inexpensive, and now it is the most commonly used route to prepare GNRs with high yield, high quality, and controllable size. The nucleation and growth of nanoparticles were conducted separately in this method [21]. The typical synthesis procedure [21, 22] is that the gold seeds prepared in advance were added to a growth solution having chemical precursors, which precipitated onto the seeds and grew into GNRs with a certain aspect ratio. During this procedure, the size of GNRs could be controlled by adjusting the molar ratio of seeds to gold salt in the growth solution and the addition of silver ions (Ag^+^) was very important to improve the yield and control the aspect ratio of GNRs [22], but there were many spherical GNPs in the product. To reduce the content of spherical GNPs, Nikoobakht et al. [23] improved the seed-mediated growth method using hexadecyltrimethylammonium bromide (CTAB) to replace sodium citrate, and GNRs with aspect ratios of 1.5–4.5 were obtained by adjusting Ag^+^ concentration in the growth solution. In this research, the GNRs with longer aspect ratios of 4.5–10 were also successfully prepared using binary surfactants CTAB and benzyldimethylhexadecylammonium chloride (BDAC) in the growth solution (Fig. 2). Moreover, researchers found that the aspect ratio and morphology of GNRs was dependent on the pH of the growth solution [24], and an impurity in CTAB was very important for GNRs formation [25].

### Seedless method

**Fig. 3:**
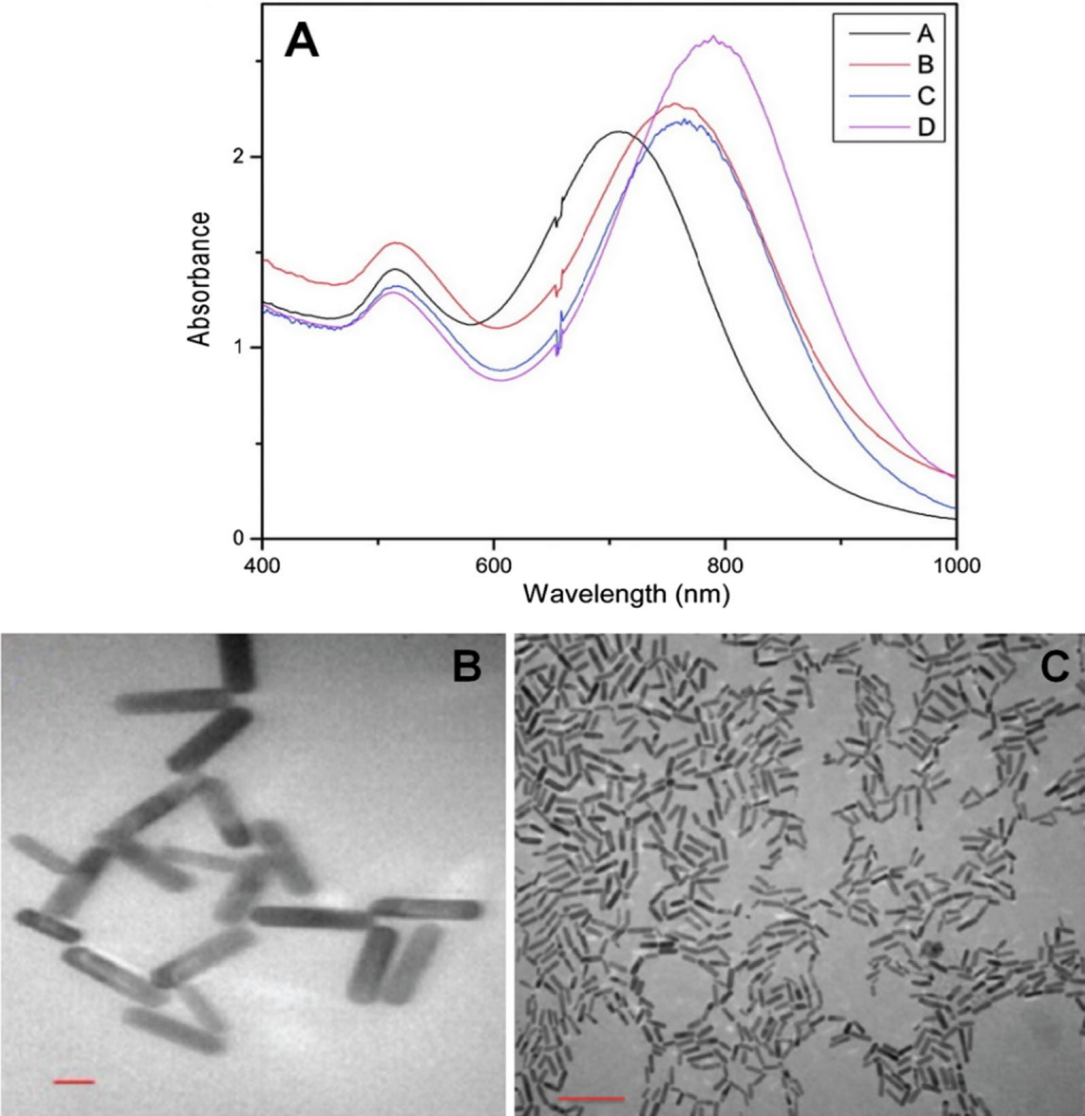
**A** UV–vis-NIR absorption spectra of growth solutions with addition of different volume of NaBH_4_ (0.01 M) at pH 2: **A** 2, **B** 5, **C** 10, and **D** 15 μL; TEM images of GNRs prepared with the seeded method (**B**) and seedless method (**C**). The aspect ratio (4.8) of the two GNRs is nearly the same. The bar indicates 20 nm for (**B**) and 100 nm for (**C**), respectively (Reproduced with permission [26]. Copyright 2012, American Chemical Society)

Unlike the seed-mediated method, the nucleation and growth of nanoparticles are in the same reaction system for this method, thus, synthesizing seeds in advance is unnecessary. Ali et al. [26] synthesized the GNRs using a one-pot seedless method. The research results demonstrated that at pH 2, the higher the concentration of sodium borohydride (NaBH_4_), the more obvious red shift effect in the longitudinal peaks of growth solutions (Fig. 3A). Furthermore, this research indicated that the GNRs synthesized with seedless method showed the same aspect ratio (4.8 ± 0.6) as those prepared by the seeded method, both by adding silver nitrate (AgNO_3_) (270 μL; 4.0 mM) to the growth solutions; however, the former exhibited a larger particle size (54.0 × 11.2 nm, Fig. 3B) compared with the latter (25.0 × 5.2 nm, Fig. 3C). Yan et al. [27] developed a modified seedless method to synthesize high quality of small GNRs ((18 ± 5 nm) × (5 ± 1 nm)) at large-scale. The LSPR band peak of the as-prepared GNRs was around 780 nm, and they exhibited excellent capabilities of photothermal therapy and PA imaging.

### Template method

**Fig. 4:**
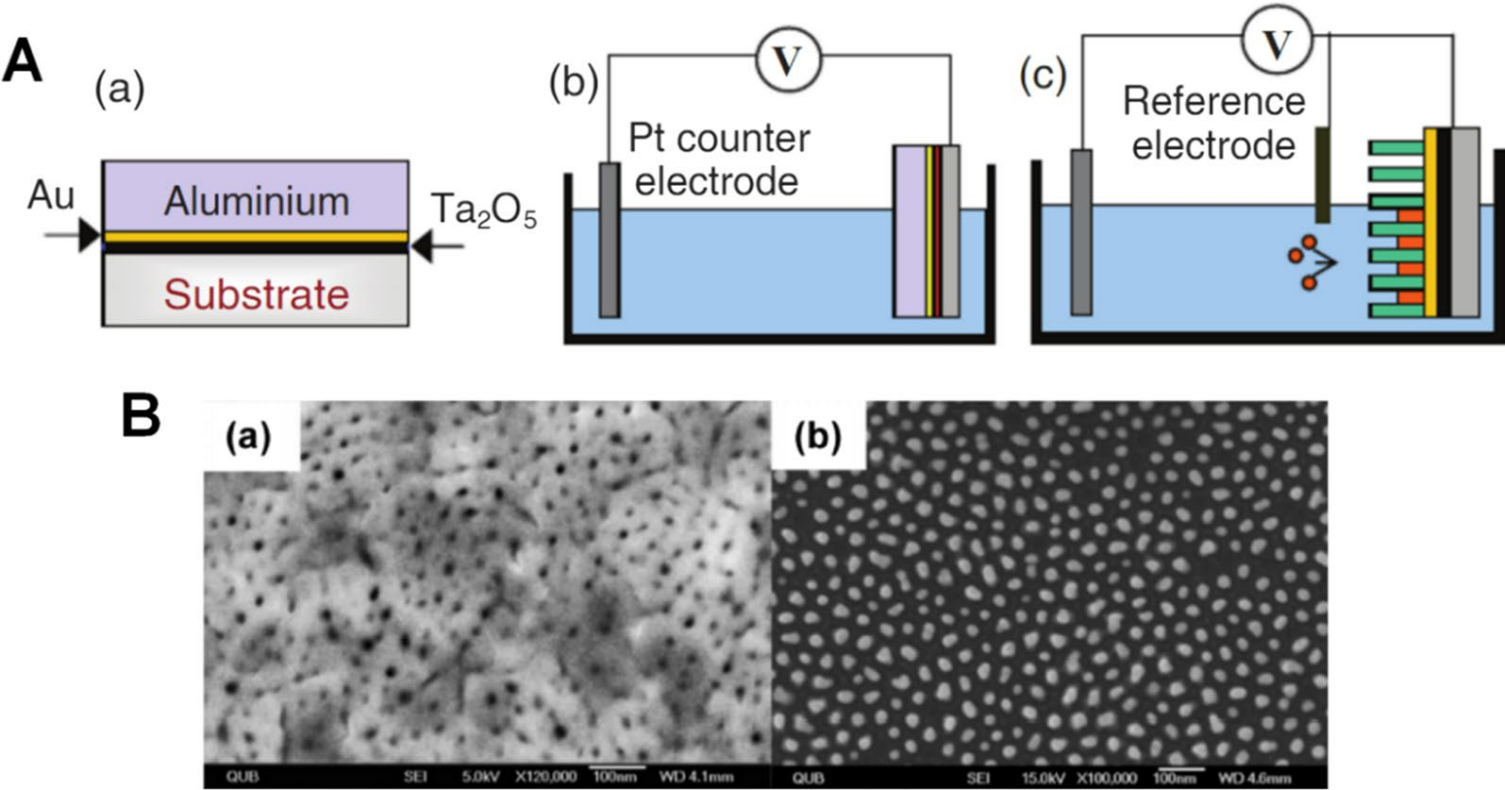
**A** Schematic of template method: (a) aluminum, gold and tantalum oxide films are deposited onto glass substrates; (b) anodization of the aluminum film is carried out using sulfuric acid; (c) GNRs are electrochemically deposited in the porous template. **B** SEM images of (a) anodic aluminium oxide template created by self-assembly and (b) arrays of GNRs (350 nm in length and 22 nm in diameter, respectively) after removal of the template by chemical etching (Reproduced with permission [30]. Copyright 2008, WILEY-VCH Verlag GmbH & Co. KGaA, Weinheim)

In the template method, porous materials with pore size ranging from nanometers to microns are used as templates to allow precursors to enter and react on the pore wall. Martin et al. [28] first introduced this method to synthesize gold nanofibrils in 1994. The desired GNRs can be synthesized using this method combined with electrochemical precipitation-, sol–gel-, and gas phase precipitation method [29, 30]. As shown in Fig. 4, during synthesis, the growth of nanorods was limited by the diameter of the pores on a film, and the length of nanorods was controlled by the amount of Au atoms deposited in pores. Thus, the aspect ratio of GNRs could be controlled by adjusting the size and length of pores and the time of electrochemical deposition [30]. However, the size of GNRs was significantly affected by environmental factors during preparation, resulting in low productivity.

Gao et al. [31] improved the template method, employing silica nanotubes as template, for large-scale growth of GNRs with low polydispersity. In this route, the selectively functionalized inner surfaces of silica nanotubes realized the selective deposition of gold inside the nanotubes, and thus the GNRs having 17 nm of diameter (aspect ratio 3.5–21) formed after the template dissolved.

### Photochemical method

**Fig. 5:**
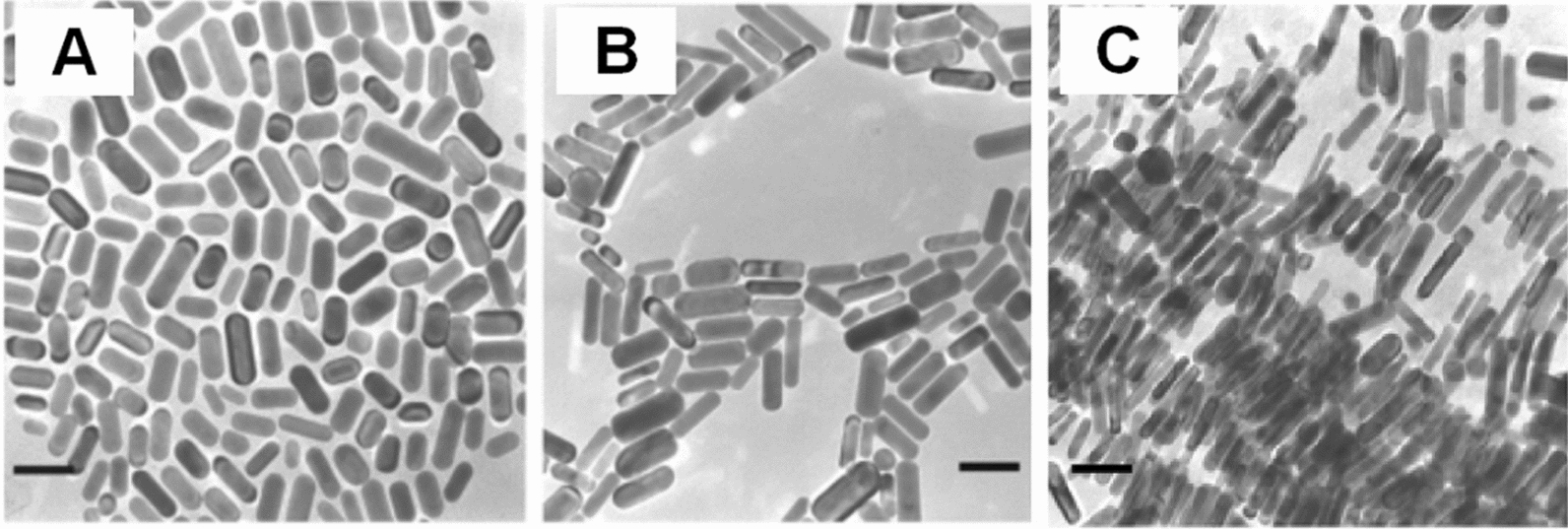
TEM images of GNRs prepared via the photochemical method with **A** 15.8, **B** 23.7, and **C** 31.5 µL of silver nitrate solution; the average aspect ratio of the GNRs is 2.8 (**A**), 3.5 (**B**), and 4.8 (**C**), respectively. The bar indicates 50 nm (Reproduced with permission [32]. Copyright 2002, American Chemical Society)

Kim et al. [32] synthesized the GNRs via a photochemical method in the presence of Ag^+^. In this method, aqueous solution of CTAB and tetradodecyl ammonium bromide as growth solution and hydrogen tetracholoaurate (HAuCl_4_·3H_2_O) as the precursor of gold were mixed. In order to loosen the micellar structure, then acetone and cyclohexane were added to the above mixture solution; afterward, AgNO_3_ aqueous solution was added and the obtained mixture solution was irradiated with UV light (254 nm) for about 30 h. The research results indicated that the synthesis of GNRs could be controlled by adjusting the Ag^+^ concentration (Fig. 5), and the higher the Ag^+^ concentration the larger the aspect ratio of GNRs, and the synthesis failed if Ag^+^ was not added. Toussi et al. [33] prepared the GNRs via the photochemical reduction of gold precursor in CTAB aqueous solution; the research results indicated that increasing of the CTAB concentration led to the obvious enhancement in the aspect ratio of GNRs.

### Electrochemical method

**Fig. 6:**
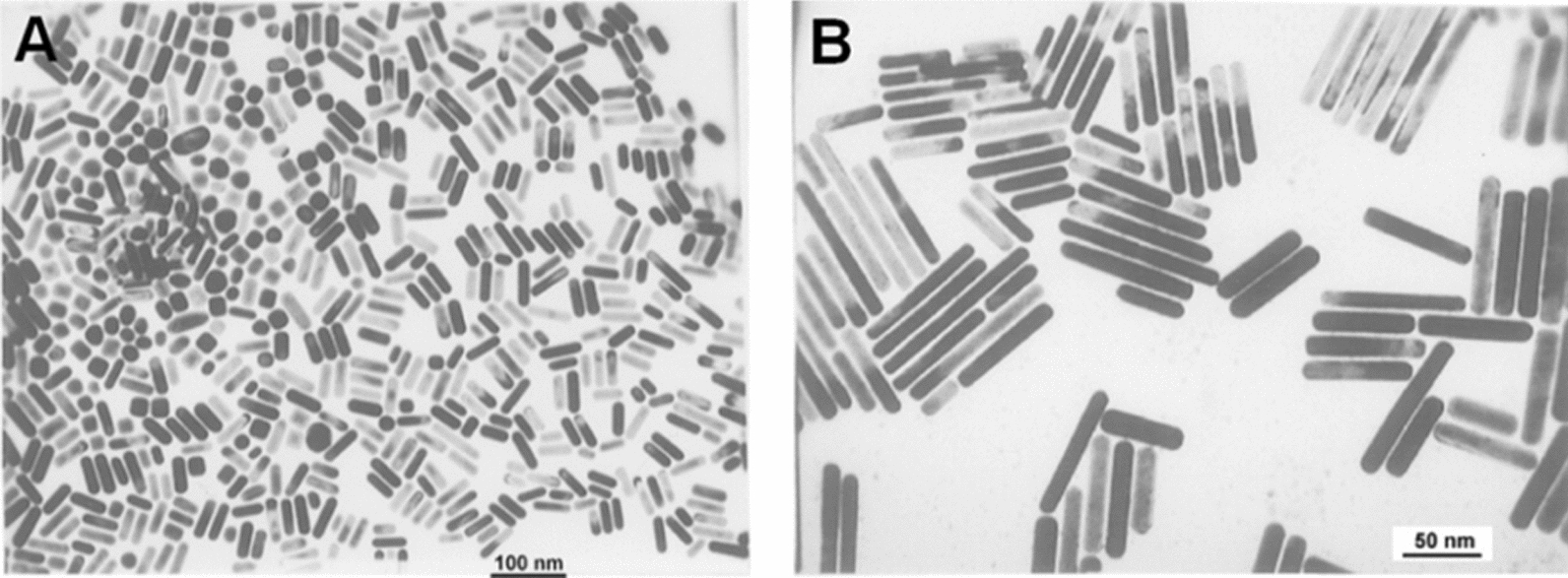
TEM images of GNRs with different mean aspect ratios synthesized via electrochemical method: 2.6 (**A**) and 7.6 (**B**) (Reproduced with permission [34]. Copyright 1997, American Chemical Society)

Electrochemical method was introduced by Yu et al. [34] to synthesize GNRs. In this method, GNRs were synthesized in an electrochemical cell using platinum (Pt) plate and gold (Au) plate as anode and cathode, respectively, which were immersed in electrolytic solution having cationic surfactant CTAB and rod-inducing cosurfactant tetraoctylammonium bromide (TCAB). The gold plate at anode lost electrons via electrolysis, which was controlled by ultrasonic temperature, to become gold ions in electrolytic solution. One portion of gold ions moved to cathode and were reduced to elemental gold, the other portion of gold ions in electrolytic solution were reduced by CTAB and turned into a midbody, then the CTAB-conjugated midbody together with elemental gold attached to the Pt plate formed the nanorods (Fig. 6). During this process, CTAB not only acted as electrolyte but also prevented nanorods from aggregation, and TCAB could induce GNRs formation. In this research, the aspect ratio of GNRs could be controlled by adjusting the current density or the CTAB/TCAB ratio. Chang et al. [35] utilized a modified electrochemical route to prepare the GNRs with average aspect ratio ranging from 1 to 7; they found that the larger the total area of immersed silver plate at the end of electrolysis the longer the obtained GNRs.

### Sonochemical method

**Fig. 7:**
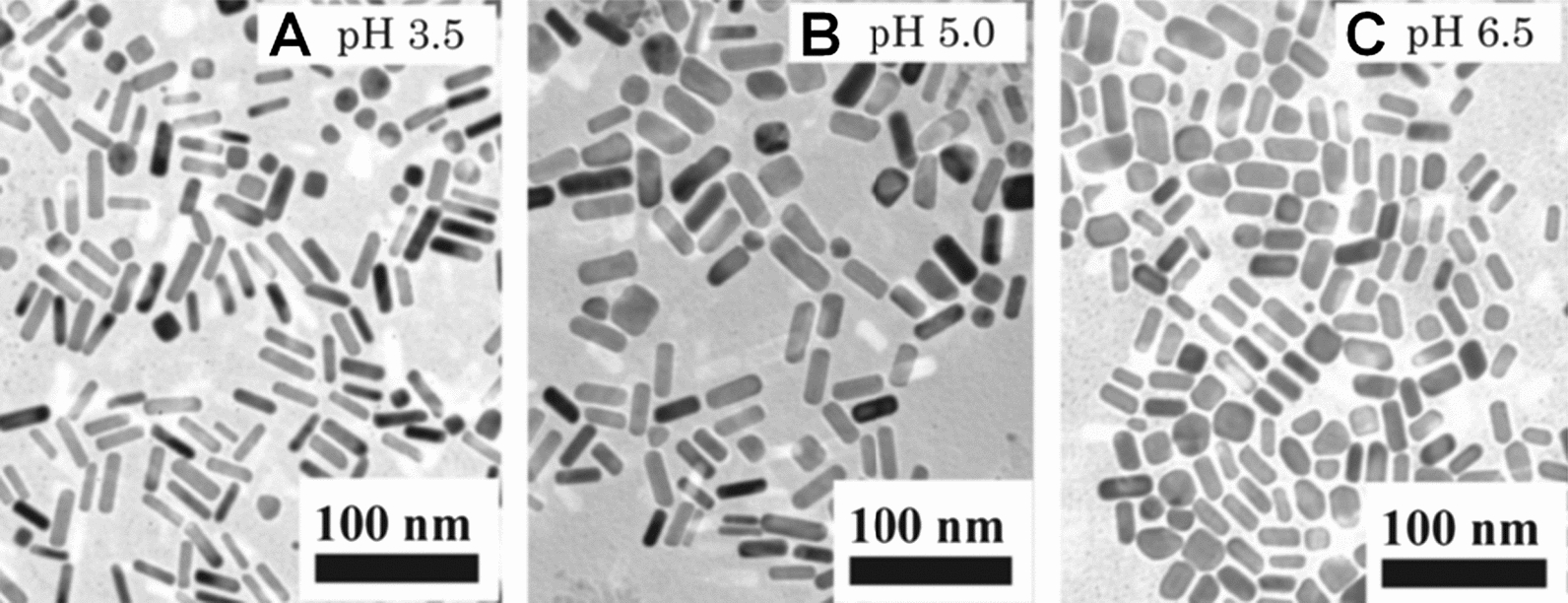
TEM images of GNRs prepared in different pH solutions with **A** pH 3.5, **B** pH 5.0, and **C** pH 6.5 after 180 min irradiation under argon (Reproduced with permission [36]. Copyright 2009, American Chemical Society)

Okitsu et al. [36] reported the synthesis of GNRs using the sonochemical method. The research demonstrated that the obtained GNRs showed less than 50 nm of size, and the average aspect ratio of GNRs decreased as the pH value of the solution increased (Fig. 7), and GNPs with irregular morphologies would be formed if the pH value of solution increased to 7.7.

Till now, the methods discussed above can achieve preparing GNRs with good uniformity and dispersity, and the size and aspect ratio of GNRs can be controlled by adjusting various experimental parameters during synthesis. However, for the potential biomedical applications of GNRs, green and more repeatable methods for GNRs preparation should be further developed.

## Toxicity reduction of GNRs

GNRs are able to maintain their colloidal stability in the presence of a certain amount of ligands on their surface, otherwise, they will aggregate irreversibly. The most common ligand on the surface of GNRs is CTAB, which acts as cationic surfactant. Although CTAB is able to significantly increase the productivity of GNRs during synthesis, the CTAB-capped GNRs show cytotoxicity owing to the positive charge of CTAB and thus induce cell necrosis in vitro [37]. Therefore, removing or replacing CTAB is an indispensable step before any further biological investigations on GNRs. The toxicity reduction of GNRs involves CTAB removal/replacement and the concomitant surface modification. Four routes, including centrifugation-washing-, phase transfer-, ligand exchange-, and surface coating method, are discussed for toxicity reduction of GNRs in this manuscript.

### Centrifugation-washing method

Centrifugation-washing method is the basic and commonly used route to remove CTAB on the surface of GNRs [38–41]. This method removes the excess CTAB on the surface of GNRs by centrifuging GNRs solution and washing GNRs with deionized (DI) water. Although this method is capable to remove most of the CTAB on GNRs, it is always accompanied by other method (e.g. phase transfer-, ligand exchange-, and surface coating method) for further CTAB removal or replacement.

### Phase transfer method

The thiol-containing ligands are commonly employed to replace the CTAB on GNRs thanks to their strong affinity to surface of Au. In a typical phase transfer procedure, the CTAB-capped GNRs were transferred from aqueous phase into an organic solvent with hydrophobic thiol-containing ligands, which could replace CTAB by being conjugated onto the surface of GNRs.

Among the thiol-containing ligands, 11-mercaptoundecanoic acid (MUA) as a thiol-containing carboxylic acid is often utilized to replace CTAB because that their carboxyl terminals could be further conjugated with biomolecules. For instance, Su et al. [42] replaced the CTAB on GNRs using MUA via a round-trip phase transfer route. The CTAB-capped GNRs aqueous solution, water-immiscible ionic liquid (IL) 1-butyl-3-methylimidazolium bis(trifluoromethanesulfonyl)imide ([BMIM][Tf2N]) and MUA/IL solution were firstly mixed and then vortex stirring was carried out, and finally the MUA-capped GNRs were obtained via ligand exchange mediated by IL. The obtained MUA-capped GNRs had improved colloidal stability and biocompatibility. Similarly, Li et al. [43] prepared the MUA-capped GNRs by a round-trip phase transfer method; the obtained MUA-capped GNRs were further modified by albumin and loaded with paclitaxel (PTX) successively for photothermo-chemotherapy of tumor model in mice. Casas et al. [44] reported that CTAB on GNRs could be replaced by MUA via a double phase transfer ligand exchange using dodecanethiol (DDT); the obtained MUA-capped GNRs kept the morphology and plasmon peak position compared with those of CTAB-capped GNRs.

Other thiol-containing ligands, such as phospholipid bilayer and partially thiolated polyamidoamine (PAMAM) dendrimer, were also reported for replacing CTAB by phase transfer method. Lee et al. [45] replaced the CTAB on GNRs using a phospholipid bilayer via a water–chloroform phase transfer route mediated by thiolated PEG. During the procedure, two different thiolated lipid molecules 1,2-dipalmitoyl-sn-glycero-3-phosphothioethanol (DPPTE) and 1,2-distearoyl-sn-glycero-3-phosphocholine (DSPC) were successively introduced to GNRs surface in chloroform and water, respectively, and thus a lipid bilayer was formed on GNRs surface owing to the hydrophobic interaction between DPPTE and DSPC. These lipid bilayer-capped GNRs showed good stability in PBS and NaCl solutions. Huang et al. [46] reported a phase transfer method for synthesizing partially thiolated polyamidoamine (PAMAM) G4.0 dendrimer-capped GNRs. They firstly obtained the MUA-capped GNRs, then the MUA-capped GNRs were further customized with partially thiolated polyamidoamine (PAMAM) G4.0 dendrimer. The synthesized dendrimer-capped GNRs demonstrated no obvious change in plasmon peak width or position.

### Ligand exchange method

This method refers to replacing CTAB on GNRs with other bicompatible ligands, and it is actually involved in the phase transfer method described above.

In this method, poly(ethylene glycol) (PEG) or PEG-based polymer is the commonly used ligand. Buckway et al. [39] removed the excess CTAB on the surface of GNRs via centrifugation and washing by DI water, and then utilized PEG to coat the GNRs. Dickerson et al. [40] reported that the excess CTAB on GNRs could be removed by that GNRs solution was centrifuged twice at a rate of 20,000×*g* for 15 min and re-dispersed in DI water, and then they used thiolated poly (ethylene) glycol (mPEG-SH) to bind to the surface of GNRs. Liu et al. [47] replaced CTAB on GNRs with polythiol PEG-based copolymer to produce an effective NIR photothermal agent. The research demonstrated that the cytotoxicity of GNRs modified by polythiol PEG-based copolymer largely decreased compared to the original CTAB-capped GNRs. Other polymers, such as polyvinyl alcohol (PVA) and polyvinylpyrrolidine (PVP), were also reported for ligand exchange of GNRs. Kinnear et al. [48] removed the majority of CTAB on GNRs using biocompatible polymer polyvinyl alcohol (PVA) as coating. The obtained PVA-GNRs showed good biocompatibility on human blood monocyte derived macrophages (MDMs). Zhao et al. [49] removed the excess CTAB on GNRs by washing with DI water, and then used polyvinylpyrrolidone (PVP) to coat the GNRs as photosensitizers for two-photon photodynamic therapy in vitro; the in vitro cellular cytotoxicity experiment demonstrated that the PVP-modified GNRs had excellent biocompatibility.

As described in phase transfer method section [42–44], thiol-containing carboxylic acids are often utilized to be conjugated with GNRs. Garabagiu et al. [41] reported that the GNRs solution was centrifuged 3 times at 10,000 rpm to remove the CTAB and then employed 3-mercaptopropionic acid (MPA) for further surface functionalization of GNRs. Dai et al. [50] utilized a place exchange reaction (carried out inside an ionic exchange resin) between CTAB-capped GNRs and MUA to replace CTAB by MUA. He et al. [51] removed the excess CTAB on GNRs by centrifugation and washing, and then added NaBH_4_ and ethanol solution of MUA to the GNRs for the formation of MUA layer on the surface of GNRs. The research results indicated that the MUA-capped GNRs showed remarkably improved biocompatibility.

### Surface coating method

Employing silica (SiO_2_) or metal–organic frameworks (MOFs) to coat GNRs is also an efficient route to improve the colloidal stability and biocompatibility of GNRs. SiO_2_ has been widely used to coat GNRs since it is able to yield products with good dispersity and biocompatibility and involves easy surface modification [52–57]. MOFs, as a new type of porous materials with unique properties, have also been widely utilized to coat GNRs to synthesize GNR@MOF core–shell structures for biomedical applications. For instance, Shang et al. [58] replaced CTAB on GNRs with MUA and then synthesized Au NR@MIL-88(A) core–shell structure as a tripe-modality imaging agent. Li et al. [59] prepared the zeolitic imidazolate framework-8 (ZIF-8)-coated GNRs with core–shell nanostructure for cancer therapy. Zeng et al. [60] reported the growth of porphyrinic MOFs on the surface of GNRs for constructing core–shell Au NR@MOFs (MOF: Zr_6_(TCPP)_1.5_ (TCPP = tetrakis (4-carboxyphenyl)porphyrin)) as a theranostic nanoplatform.

The ligand or coating utilized for toxicity reduction of GNRs discussed above are summarized in Table 1.

**Table 1:** Ligand or coating utilized for toxicity reduction of GNRs discussed in this manuscript

Ligand or coating utilized	Refs.
PEG or PEG-based polymer	[39, 40, 47]
PVA	[48]
PVP	[49]
MPA	[41]
MUA	[42–44, 50, 51, 58]
Phospholipid bilayer	[45]
Partially thiolated polyamidoamine (PAMAM) dendrimer	[46]
SiO_2_	[52–57]
MOFs	[58–60]

## GNRs as radiosensitizers in radiation therapy

Nanostructures with high-Z are ideal radiosensitive materials for radiation enhancement. Taking the X-ray absorption by materials as an example, the X-ray absorption coefficient (μ), incident X-ray energy (E), and atomic number (Z) of materials are based on the following relationship: μ = ρZ^4^/(AE^3^), where ρ and A are the density and atomic mass of materials, respectively [61]. Nanostructures with high-Z are able to enhance X-ray absorption in local tissues and effectively release low-energy electrons to generate more free radicals, and the energy (deposited efficiently by free radicals and electrons) can cause damage to DNA [62]. Element Au with high-Z (Z = 79) make GNRs widely used in external- and internal radiation therapy. GNRs as radiosensitizers in radiation therapy is divided into two parts (external- and internal radiation therapy) for discussion in this manuscript.

### GNRs as radiosensitizers in external radiation therapy

GNRs are able to cause sensitization with external radiation (e.g. X-rays) in vitro and in vivo. GNRs are often modified with PEG or a layer of silica to improve their colloidal stability in radiation therapy. Besides, if GNRs or GNRs-based nanocomposites are conjugated with targeting ligands, such as specific antibody and peptide, their accumulation in tumor site will increase greatly, and thus their radiation therapy efficacy will be improved.

**Fig. 8:**
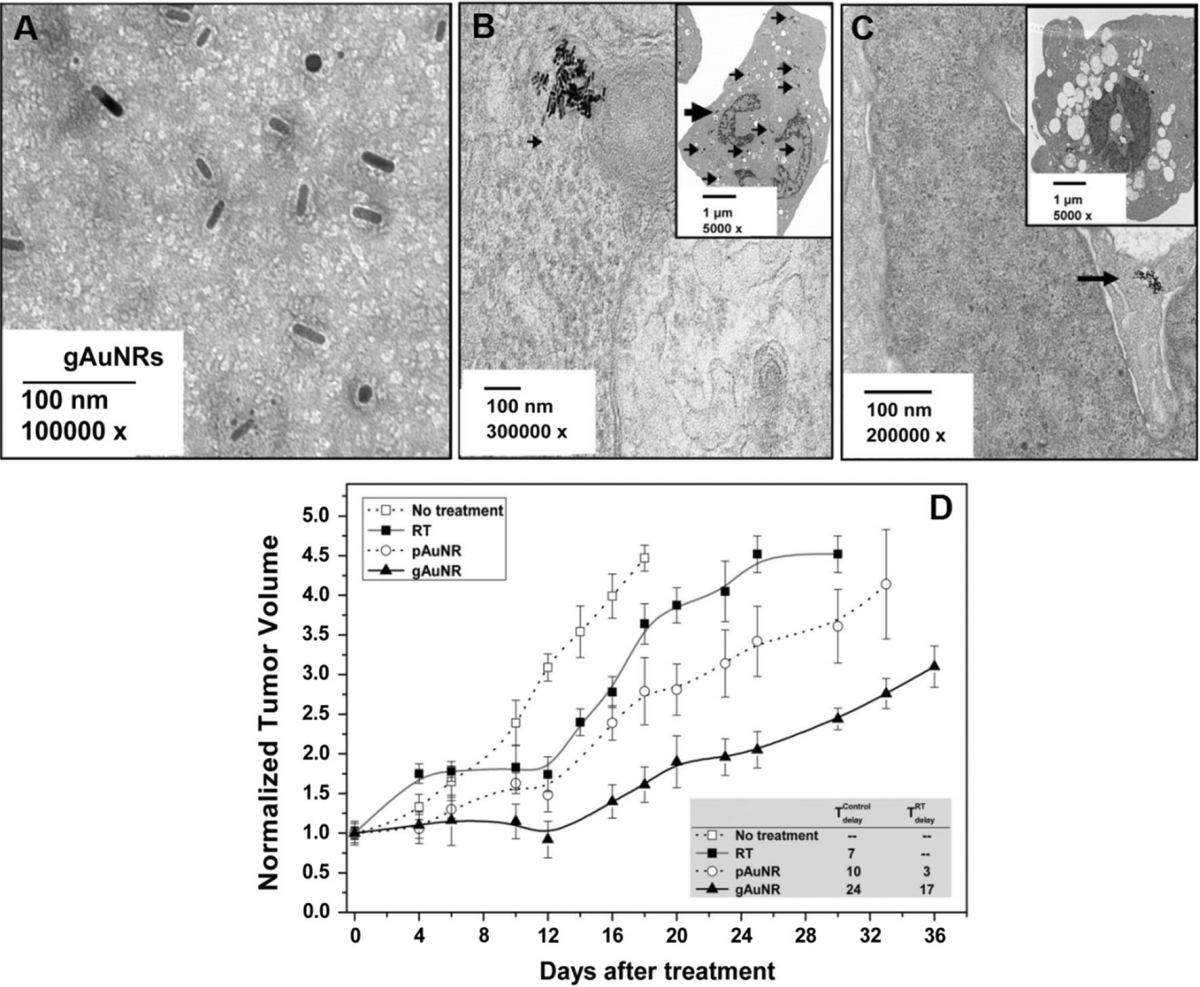
TEM image of gAuNRs (**A**); TEM images of PC3 cells treated with gAuNRs (**B**) and pAuNRs (**C**) for 24 h of incubation. Arrows point to AuNRs; **D** Tumor-growth in nude mouse xenograft models with and without treatment by AuNRs before radiation therapy (RT) with 6 MV beam. The inside is the table showing tumor-growth delays, comparing the groups that given either gAuNR and RT (filled triangle) or pAuNR and RT (open circle) with the groups that given only RT (filled square) and no treatment (open square) (Reproduced with permission [64]. Copyright 2015, Elsevier Ltd)

Xu et al. [63] prepared arginine-glycine-aspartate (RGD)-conjugated GNRs with a layer of silica (RGD-GNRs) to enhance the radiation therapy of melanoma cells. The RGD-GNRs could be internalized into A375 melanoma cells by integrin αvβ3-receptor mediated endocytosis. The following trend of radiosensitizing effect of treatment groups was demonstrated in this research: RGD-GNRs plus radiation (X-ray) > radiation alone > RGD-GNRs > GNRs, attributed to that RGD-GNRs could downregulate αvβ3 expression. The cell cycle of A375 cells treated by GNRs, RGD-GNRs, radiation alone, and RGD-GNRs plus radiation was arrested at the G2/M phase of 35% ± 2.65%, 36.14% ± 0.35%, 40.9% ± 0.35%, and 46.5% ± 1.2%, respectively. Zhao et al. [53] investigated the radiosensitizing effect of RGD-conjugated and mesoporous silica-coated GNRs (GNRs@mSiO_2_-RGD) multifunctional nanoprobe in MDA-MB-231 triple-negative breast cancer (TNBC) cells in response to megavoltage (MV) radiation. The research results demonstrated that GNRs@mSiO_2_-RGD plus radiation had a synergistic effect of increasing the population of MDA-MB-231 cells in the G2/M phase and the proportion of apoptotic cells. Wolfe et al. [64] synthesized goserelin-conjugated GNRs (gAuNRs) for radiation therapy of prostate cancer. The targeting ligand goserelin promoted the internalization of GNRs within prostate cancer (PC3) cells and enhanced the sensitization of prostate tumors to MV radiation therapy in vivo. In this research, the gAuNRs, whose TEM image was shown in Fig. 8A, accumulated inside the PC3 cells at higher concentration than PEGlated GNRs (pAuNRs) (Fig. 8B, C). The quantitative inductively coupled plasma-mass spectrometry (ICP-MS) data showed that the concentration of gAuNRs within cells reached the maxima at 24 h of post-incubation, which was 5 times greater than pAuNRs. Moreover, the mice treated with gAuNR and radiation therapy showed obviously enhanced tumor-growth delays, but not delay with radiation therapy alone (Fig. 8D).

Khoo et al. [65] also reported goserelin-conjugated GNRs for radiosensitization of prostate cancer using erbium (Er)-filtered X-ray. The research demonstrated that the combined treatment of goserelin-conjugated GNRs and Er-filtered X-ray irradiation had more obvious tumor growth inhibition effect in mice bearing prostate cancer xenografts tumors than radiation treatment only. Chang et al. [66] synthesized two types of peptide (RGD and ACPP)-coated core–shell Au@Se nanocomposites for synergistic radiochemotherapy using the radiosensitization effect of GNRs and the antitumor activity of Se NPs. The research exhibited that the nanocomposite treatment plus X-ray could induce apoptosis of A375 melanoma cells and trigger intracellular ROS overproduction, and could further suppress the tumor growth in vivo. Ma et al. [67] investigated the effect of the shape of gold nanostructures on the efficiency of X-ray radiation therapy. They prepared gold nanomaterials with similar average size of about 50 nm but different shapes, including spherical GNPs, gold nanospikes (GNSs), and GNRs, and then coated them with PEG molecules. The research demonstrated that these gold nanomaterials incubated with KB cells for 24 h showed cellular internalization efficiency in the order of GNPs > GNSs > GNRs, and the radiosensitization effect of these gold nanomaterials exhibited the same order as that of cellular internalization efficiency, and the sensitization enhancement ratios (SERs) corresponding to the treatments of the above gold nanostructures (GNPs, GNSs, and GNRs) were 1.62, 1.37, and 1.21, respectively. These results indicated that the radiosensitization effect of gold nanostructures was determined by the internalized amount of gold atoms. The studies on radiosensitization mechanism showed that gold nanostructures could induce ROS level and cell cycle redistribution, and thus to improve the radiosensitization effect.

**Fig. 9:**
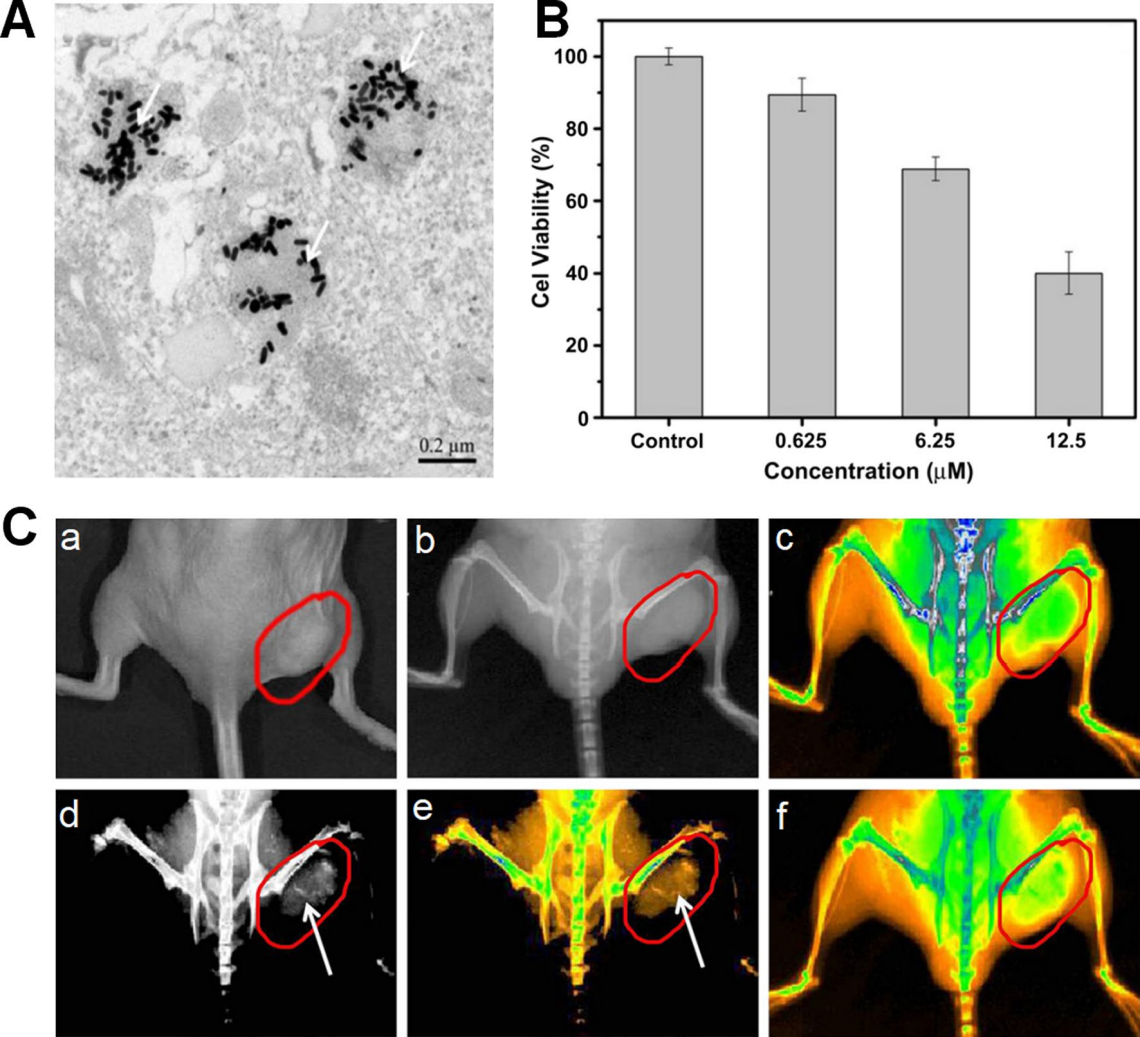
**A** The thin-section TEM image of MGC803 cells incubated with 50 mM of GNR-SiO_2_-FA for 2 h; **B** The viability of MGC803 cells incubated with 100 mL of GNR-SiO_2_-FA with various concentration for 24 h under 6 Gy of X-ray irradiation; **C** Real-time in vivo X-ray images after intravenous injection of GNR-SiO_2_-FA in nude mice at different time points: (a) Photograph of the tumor tissue; (b) X-ray image at 0 h; (c) X-ray image at 0 h (in color); (d) X-ray image at 12 h, (e) X-ray image at 12 h (in color); (f) X-ray image at 24 h (in color) (Reproduced with permission [19]. Copyright 2011, Elsevier Ltd)

The high NIR photothermal conversion efficiency and strong X-ray attenuation abilities of GNRs make them as photothermal agents and radiosensitizers in synergistic treatment of photothermal therapy and radiation therapy. The mechanism of this synergistic treatment is the following: hyperthermia increase the temperature of local tumor tissues, soften blood vessels, promote blood circulation and oxygen transport in tumor microenvironment, improve the sensitivity of hypoxic tumor cells to radiation, and inhibit the repair of radiation injury of tumor cells. The combination of radiation therapy with photothermal therapy using GNRs can reduce radiation dose and improve radiation therapy efficacy. Huang et al. [19] synthesized folic acid-conjugated silica-coated GNRs (GNR-SiO_2_-FA) to selectively target MGC803 gastric cancer cells and enhance the effect of radiation therapy and photothermal therapy on MGC803 cells. The TSPR- and LSPR wavelength of the obtained GNR-SiO_2_-FA was 527 nm and 732 nm, respectively. The thin-section TEM image of MGC803 cells treated by GNR-SiO_2_-FA indicated that GNR-SiO_2_-FA could be endocytosed via the folate receptor-mediated path in endosomes (Fig. 9A). Under X-ray irradiation, the viability of MGC803 cells treated by GNR-SiO_2_-FA for 24 h decreased as GNR-SiO_2_-FA concentration increased (Fig. 9B). The GNR-SiO_2_-FA demonstrated strong X-ray attenuation for in vivo X-ray/CT imaging, and the tumor targeting ability of GNR-SiO_2_-FA was exhibited by the real-time in vivo X-ray images in mice (Fig. 9C). This GNR-SiO_2_-FA could be utilized for X-ray/CT imaging-guided and targeted radiation therapy and photothermal therapy.

**Fig. 10:**
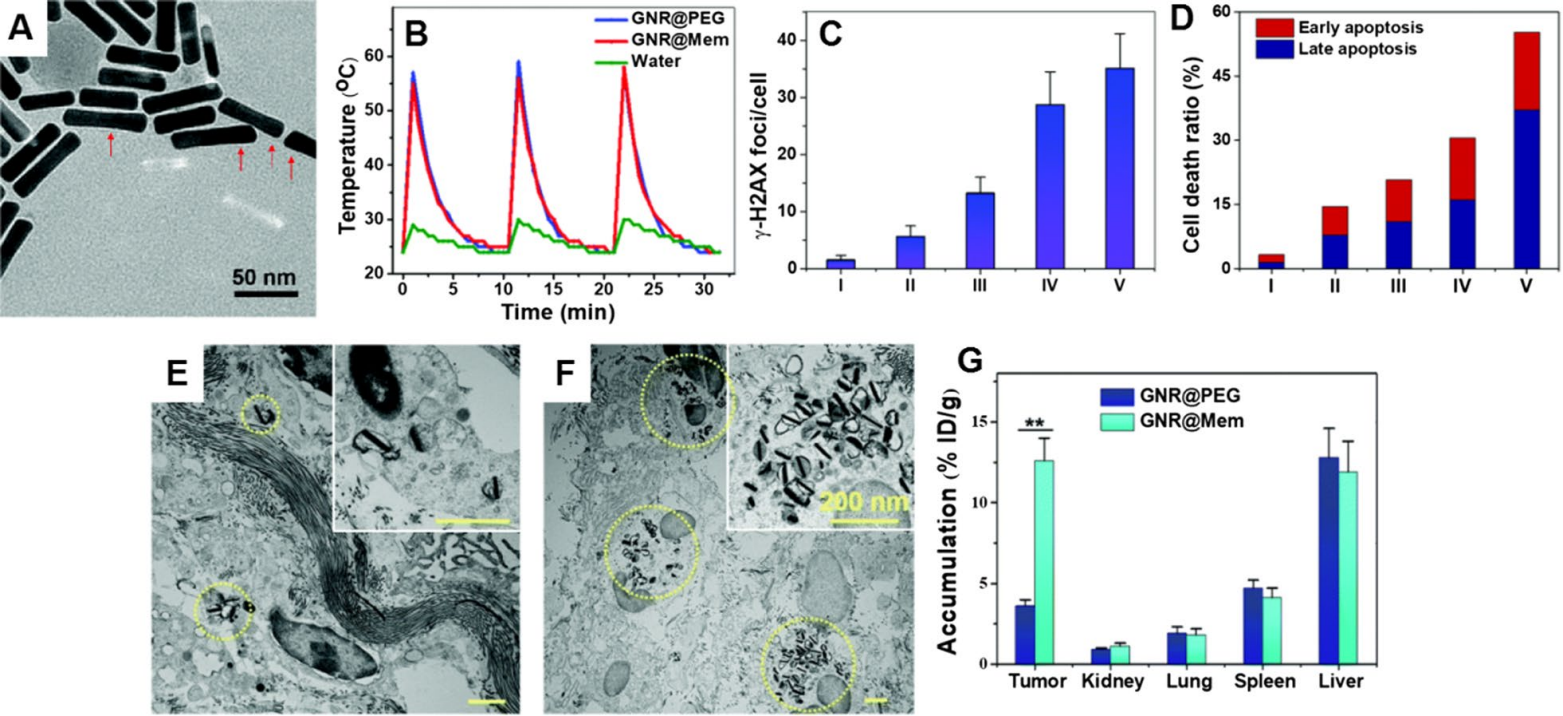
**A** TEM image of GNR@Mem; **B** temperature of GNR@Mem dispersion as a function of irradiation time under 980 nm light at 0.5 W/cm^2^ for 1 min (the GNR concentration was 100 mg/mL); Quantitative analysis of **C** ɣ-H2AX foci density, and **D** cell apoptosis and necrosis ratio in different treatment groups (I—Control, II—4 Gy, III—GNR@PEG + 4 Gy, IV—GNR@Mem + 4 Gy, V—GNR@Mem + 4 Gy + NIR; the GNR concentration was 20 mg/mL); TEM images of the tumors in mice treated by **E** GNR@PEG and **F** GNR@Mem (scale bars are 200 nm); **G** Tissue distribution of GNRs 24 h after i.v. injection. (Reproduced with permission [70]. Copyright 2020, Royal Society of Chemistry)

Li et al. [68] reported that the radiosensitizing effect of RGD-conjugated GNRs (RGD-GNRs) combined with NIR irradiation was greater than that of RGD-GNRs alone in A375 cells, and thus the apoptosis of cells increased and the proportion of cells in the more radioresistant S phase decreased. Movahedi et al. [69] investigated the photosensitivity and radiosensitivity of folate-conjugated GNRs (AuNR-FA) on KB nasopharyngeal carcinoma cells. ICP-MS analysis confirmed that the uptake of AuNR-FA in KB cells was higher than that of GNRs. MTT assay showed that the combination of photothermal therapy and radiation therapy using AuNR-FA significantly reduced the viability of KB cells. Sun et al. [70] fabricated the GNRs coated with human oral squamous KB cancer cell membrane (GNR@Mem), as shown in Fig. 10A, for targeting KB cancer cells. The obtained GNR@Mem exhibited high photothermal transfer efficiency and superb stability under 980 nm NIR light (Fig. 10B), as well as high radiosensitizing ability. Compared with the control groups, the combination treatment of X-ray and NIR light irradiation improved the therapeutic effect, demonstrated in the number of ɣ-H2AX foci (Fig. 10C) and cell apoptosis and necrosis ratio (Fig. 10D). This research showed that the GNR@Mem suppressed the xenograft KB tumor growth under the irradiation of NIR light and X-ray. In addition, the TEM images of tumors of mice (Fig. 10E, F) and distribution of GNRs in tumor 24 h post-injection (Fig. 10G) both indicated that more GNRs were accumulated in the tumor of GNR@Mem group than that of GNR@PEG group, demonstrating the targeting ability of the cancer cell membrane on GNRs.

Gamma rays generated by machines outside the body were also utilized to study the external radiation therapy application of GNRs. Masood et al. [71] used the GNR-SphK siRNA nanocomposite [GNRs loaded by short interfering RNA (siRNA) targeting the anti-apoptotic sphingosine kinase (SphK1) gene] to induce radiation sensitization in head and neck squamous cell carcinoma (HNSCC) tumor in mice. The research demonstrated that GNR-SphK siRNA nanocomposite could efficiently deliver SphK siRNA to HNSCC cells and sensitize the tumor to radiation from a gamma cell 40 irradiation machine with ^137^Cs source. Compared with control group (GNR-GFP siRNA plus radiation treatment), the GNR-SphK1 siRNA plus radiation treatment group resulted in over 50% tumor regression. Furthermore, this GNR-SphK siRNA nanocomposite could inhibit the tumor growth significantly with radiation at low dosage of 5× lower than that utilized in clinical radiation therapy. Koosha et al. [72] reported the PEG-coated AuNR@MS nanostructure (GNRs were coated by mSiO_2_) for radiation therapy study. The research found that AuNR@MS nanostructures at high concentrations showed obvious radiosensitization effect on CT-26 cells under radiation of gamma ray (4 Gy) from a cobalt 60 (Co-60) machine.

### GNRs as radiosensitizers in internal radiation therapy

**Fig. 11:**
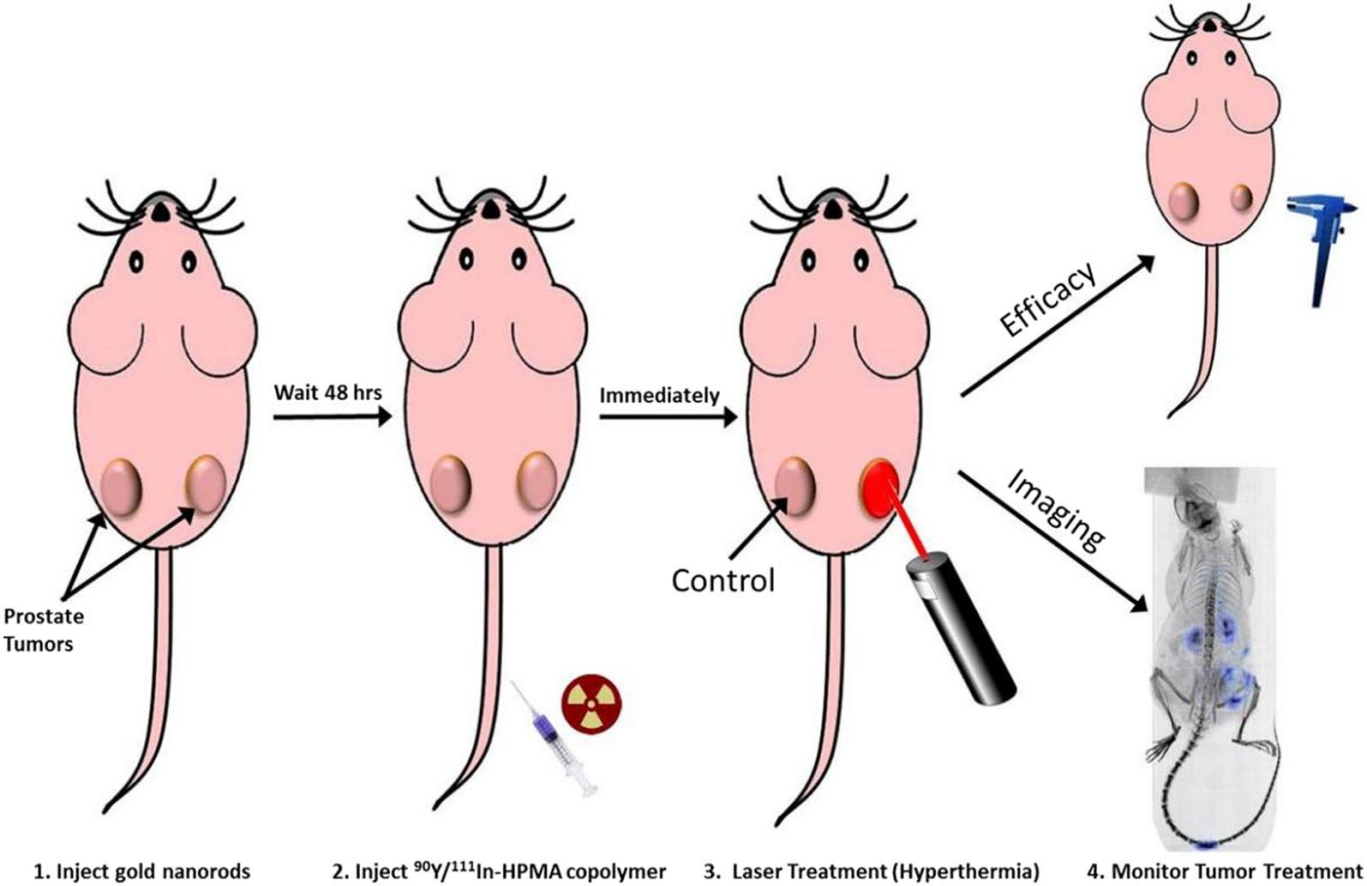
Methodology of the combination of radiation therapy and hyperthermic treatment for prostate tumor-bearing mice (Reproduced with permission [39]. Copyright 2014, Elsevier Ltd)

GNRs were also labeled by various radionuclides, such as ^90^Y and ^125^I, for internal radiation therapy of cancer. Buckway et al. [39] synthesized PEGylated GNRs (PEG-GNRs) to achieve hyperthermia; they also used HPMA copolymers labeled with ^111^In and ^90^Y for SPECT imaging and radiation therapy investigations, respectively (Fig. 11). The research demonstrated the potential of hyperthermia mediated by GNRs in sensitizing tumors to radiation therapy and the combination of photothermal therapy and internal radiation therapy was effective in DU-145 prostate tumor-bearing mouse model. Gao et al. [73] utilized folic acid-conjugated silica-coated GNRs (GNRs@SiO_2_-FA) in combination with iodine-125 (^125^I) seeds implantation for radiation therapy of cancer cells. They verified that the concentration of GNRs@SiO_2_-FA smaller than 40 µg/mL was safe for biological activity of hepatocellular carcinoma cells (HepG2) and GNRs@SiO_2_-FA could enter the cytoplasm via endocytosis. The ^125^I seeds implantation were used to irradiate cells in this research, and the results demonstrated that the combination of GNRs@SiO_2_-FA as radiosensitizer with ^125^I seeds effectively induced apoptosis in cells by increasing the protein expression of Bax and caspase-3, and this combination also inhibited the protein expression of Bcl-2 and Ki-67.

The reports on GNRs as radiosensitizers in radiation therapy discussed above are summarized in Table 2.

**Table 2:** GNRs as radiosensitizers in radiation therapy

Nanocomposite	Cell line	Tumor model	Radiation/radiation source	Refs.
GNRs@mSiO_2_-RGD	MDA-MB-231	Orthotopic transplantation nude mice models with human TNBC	X-ray	[53]
RGD-GNRs	A375	/	X-ray	[63]
Goserelin-conjugated GNRs	PC3	Subcutaneous mice models with prostate cancer	X-ray	[64]
Goserelin-conjugated GNRs	PC3	Swiss nude mice bearing subcutaneous human prostate cancer	X-ray	[65]
RGD- and ACPP coated core–shell Au@Se nanocomposite	A375	Subcutaneous nude mouse models with A375 cancer	X-ray	[66]
PEG-coated GNRs	KB	/	X-ray	[67]
GNR-SphK1 siRNA	SCC-15; USC HN-1	Male Balb/C athymic mice bearing USC HN-1 xenografts	Cs-137 gamma ray	[71]
AuNR@MS nanostructure	CT-26 cells	/	Co-60 gamma ray	[72]
PEG-GNRs	/	Athymic Nu/Nu female mice bearing DU-145 prostate tumor	^90^Y	[39]
GNRs@SiO_2_-FA	HepG2	/	^125^I	[73]

## Challenges and prospects

As a type of high-Z nanostructures, GNRs have attracted much attention in radiation therapy researches owing to their various advantages, including good biocompatibility, good radiation sensitization, unique optical properties, simple synthesis, controllable size and aspect ratio, and easy surface modification. GNRs can also serve as excellent photothermal sensitizers and CT/PA dual-model contrast agents. Therefore, more and more researches on GNR-based imaging-guided radiation therapy/photothermal therapy have been emerging. Although GNRs have shown great potential in radiation therapy, many problems should be resolved before their clinical application, especially the GNRs-based radiosensitizers with high performance and good bicompatibility should be further developed.

1. GNRs with controllable size and morphology can be prepared using the current methods by adjusting the synthetic conditions, however, detailed and systematic investigations on the effects of experimental parameters and development of better synthesis systems should be conducted, which may make the preparation of GNRs more professional and controllable.
2. The existing methods for CTAB removal/replacement are often complicated and time-consuming, thus, how to improve the treatment process and equipment for seeking a more convenient way should be pay more attention in the future research. At the same time, CTAB on GNRs usually cannot be completely removed by one method, thus, a combination of multiple methods with complementary advantages should be utilized to improve the toxicity reduction effect of GNRs.
3. The radiation sensitization of GNRs is affected by many parameters, including size and surface modification of GNRs, the utilized cell lines, the type of radiation and the radiation dose, thus, in order to construct an optimized composite system based on GNRs for radiation therapy, more researches on GNRs- and radiation- and cell line related parameters for radiation sensitization should be conducted systematically.
4. During clinical application, the accumulation of GNRs in vivo may lead to long-term toxicity, therefore, biocompatible GNRs with a suitable size should be prepared to eliminate or reduce the GNRs accumulation after cancer treatment. GNRs with a size of smaller than 5 nm demonstrate better penetration into tumor and could be rapidly eliminated via renal system. In addition, the biodistribution, toxicity, and pharmacokinetics of GNRs in radiation therapy should be systematically investigated.
5. Since the radiation dose in radiation therapy should be kept as low as possible to protect patients from risks caused by ionizing radiation, the GNRs-sensitized radiation therapy could be combined with other therapies (e.g. photothermal therapy, photodynamic therapy, and chemotherapy) to reduce the radiation dosage while achieving the maximum therapy efficacy.

## Conclusions

In summary, the research progress regarding the synthesis and toxicity reduction as well as radiation therapy application of GNRs are discussed in this review. GNRs are a type of promising theranostic agents in imaging-guided cancer radiation therapy although there are difficulties and great challenges before their clinical application.
